# Sexual Debility in Man

**Published:** 1903-10

**Authors:** 


SEXUAL DEBILITY IN MAN.—By F. R. Sturgis, M. D., formerly Clinical Professor of Venereal Diseases in the Medical Department of the University of the City of New York, etc. 381 pages, illustrated. Price, $3.00. Published by E. B. Treat & Co., 241-3 West Twenty-second Street.

In getting out this work the author seems to be actuated less by a desire to theorize on the statistics of others than to state to the medical public some conclusions which an exceptionally large venereal practice has forced upon him. He combats the very generally accepted statements—originated largely by "quacks," patent medicine advertisers, which have insidiously crept into the minds of many of our more enlightened medical men—that masturbating in childhood and youth must necessarily be followed by mental and bodily decay, etc.

In discussing the castration of masturbating lunatics, the author believes in its propriety in certain well selected cases.

He has succeeded in bringing enough clinical evidence forward to justify the separation of spermatorrhea and pollutions into two distinct diseases, and to correct the absurd idea that the sufferer from the former is irrevocably doomed to sexual decay.

The illustrations—a series of nine microscopic plates representative of normal and pathological semen—were prepared from nature by Dr. Sondern, of New York, and are excellent studies.

